# Feasibility and flexibility of a novel multi-dose level avoidance reirradiation technical methodology in recurrent head and neck cancer

**DOI:** 10.1093/bjrcr/uaae020

**Published:** 2024-07-03

**Authors:** Thomas Hague, Rikki Lad, Kevin Chiu

**Affiliations:** Department of Radiotherapy, Mount Vernon Cancer Centre, Northwood HA6 2RN, United Kingdom; Department of Radiotherapy, Mount Vernon Cancer Centre, Northwood HA6 2RN, United Kingdom; Department of Clinical Oncology, Mount Vernon Cancer Centre, Northwood HA6 2RN, United Kingdom

**Keywords:** recurrent head and neck cancer, reirradiation, multidose avoidance, reirradiation planning, medical physics

## Abstract

Reirradiation in recurrent head and neck cancer presents a considerable clinical challenge in radiation oncology. Though technically feasible due to advanced treatment delivery and planning techniques, confidence in delivering such treatments is not universal and patient selection is critical. Radiotherapy planning in reirradiation cases presents a complex technical challenge owing to the often-considerable overlap of dose from a patient’s first treatment plan. This technical note describes three clinical case studies of recurrent head and neck cancer and the technical details of how their multidose level reirradiation was planned. Each patient had confirmed recurrence of squamous cell carcinoma and was referred for reirradiation to a previously irradiated area. The clinical details for each patient are provided before a detailed description of the treatment planning methodology is presented, which specifies how to approach such complex overlapping treatment volumes. The patient outcomes are described and a discussion is presented outlining the clinical challenges associated with these cases and the variables that must be accounted for when considering patients for potential reirradiation.

## Introduction

Reirradiation in locoregional recurrent head and neck cancer is one of the most complex challenges in radiation oncology. Careful multidisciplinary decision-making and detailed patient consultation on the additional risks are both crucial. Although studies have shown the feasibility of various head and neck reirradiation regimens,^1–6^ the confidence of delivering reirradiation is not universal. The 2022 Royal College of Radiologists Head and Neck Consensus states that it is not appropriate for reirradiation to be delivered by all head and neck centres in the United Kingdom.^7^ The pattern of locoregional recurrence in head and neck cancer is heterogeneous, and patient selection for reirradiation is crucial.^8^^,^^9^

Intensity modulated radiotherapy (IMRT) and its rotational form volumetric modulated arc therapy are both well-established standard treatment techniques for head and neck radiotherapy, generating highly conformal dose distributions which permit improved sparing of organs at risk (OARs) compared to conventional techniques. With the increased usage of IMRT in head and neck cancer in the past decade, patients being considered for reirradiation present a technical treatment planning challenge, as the complexity of an IMRT distribution ensures that a reirradiation treatment plan can no longer be achieved through traditional techniques such as field matching and junctioning. The complex dose distributions of IMRT plans are characterized by regions of steep dose gradients and a greater proportion of low dose (termed the “low dose bath”) compared to 3D-conformal techniques, and this pattern of dose deposition contributes to treatment toxicity such as xerostomia and dysphagia.^10^ Reirradiation cases therefore require a more rigorous approach that accounts for the potential overlap of two highly complex dose distributions.

This technical note describes a technique for the treatment planning of head and neck cancer reirradiation cases and provides case studies of three highly selected clinical cases of recurrent oral cavity cancer post-primary surgery and adjuvant radiotherapy, for which the technique has been used.

## Clinical cases

### Case 1

A 55-year-old male with squamous cell cancer (SCC) of the oral tongue had a pathological (p)T2N0M0 disease following a hemi-glossectomy and selective ipsilateral neck level 1-4 dissection. Ipsilateral neck dissection was performed as the primary disease was deemed to be lateralized with more than 10 mm away from the midline radiologically. Post-operative pathology showed a moderately differentiated 33 mm SCC with 9 mm depth of invasion. The deep margin was 3 mm. Lymphovascular and perineural invasions were present. In total, 30 nodes were dissected but showed no evidence of malignancy. He underwent adjuvant radiotherapy comprising 60 Gray (Gy) in 30 fractions (f) to the tongue primary only. Within 2 months after completion of the adjuvant radiotherapy, he developed regional recurrence in the ipsilateral level 1b and contralateral level 2b nodes. The patient underwent salvage excision of the ipsilateral node and surrounding structures, as well as a modified contralateral level 1-5 dissection. The post-salvage pathology showed a 30 mm ipsilateral level 1b node with extranodal extension (ENE). The contralateral neck dissection yielded 3 positive nodes out of a total of 47, with the largest node measuring 27 mm also with ENE (pN3b). He proceeded to have additional radiation up to 65 Gy in 30f to the bilateral neck, with concomitant cisplatin.

### Case 2

A 37-year-old female had a pT2N3bM0 lateral tongue SCC following a partial glossectomy and ipsilateral neck dissection. The first post-operative pathology showed a well-differentiated 26 mm SCC that was 8 mm deep. There was perineural invasion, and the deep and inferior margins were both 4 mm. Of the 24 total dissected nodes, there were 2 positive nodes with ENE. The patient underwent adjuvant 65 Gy in 30f radiotherapy to the primary site and ipsilateral neck, with concomitant cisplatin. However, recurrence was detected in the non-irradiated contralateral level 4 neck, 9 months after completion of the post-operative chemo-radiotherapy. Modified contralateral neck dissection was performed and the pathology showed a 15 mm malignant node with ENE, out of a total of 12 nodes. Further post-operative radiotherapy up to 65 Gy in 30f with cisplatin was recommended on the provision of avoiding the previously irradiated area.

### Case 3

A 62-year-old lady first had a pT3N0M0 tongue SCC after undergoing partial glossectomy and ipsilateral neck dissection. The initial post-operative pathology showed a poorly differentiated SCC 36 mm in length and 14 mm deep. The primary tumour was deemed to be a clinically (c)T2N0M0 prior to surgery, hence ipsilateral dissection only. The closest margin was 4 mm posteriorly. Perineural invasion was present. None of the 24 nodes dissected were involved (pN0). The patient underwent post-operative radiotherapy to the primary site only with 60 Gy in 30f. A contralateral level 1b recurrence was diagnosed 2 years later which was managed with contralateral neck dissection. The node measured pathologically 35 mm with pENE. The patient was then treated with reirradiation up to 66 Gy in 33f with cisplatin.

## Materials and methods

As the department standard of care, head and neck patients are immobilized using a five-point thermoplastic shell. Planning CT datasets are acquired using a Toshiba Aquilion or Siemens Definition wide-bore CT at 2 mm slice thickness. Localization is achieved by means of a medial and two lateral ball-bearings placed on the shell. Additional MR images are imported into the treatment planning system (TPS) and co-registered to aid target volume delineation. Patients are planned and treated using the RapidArc technique delivered by up to 2.5 treatment arcs. Treatment is inversely planned using the optimizer within the Eclipse TPS (v15.1) and delivered via the Varian Aria record and verify system (v15). The Eclipse TPS uses the anisotropic analytical algorithm for dose calculation. Planning target volumes (PTVs) that extend into the buildup region or beyond into air are cropped to within 5 mm of the skin surface to avoid fluence boosting during optimization. All patients have IGRT in the form of planar kV imaging using the Varian on-board imaging system with CBCTs acquired on fractions 1-3 and weekly. All plans undergo an independent monitor unit (MU) check prior to treatment using the PTW Diamond software.

### Treatment planning a reirradiation case

The following sections describe the method used for treatment planning a reirradiation case. The accompanying figures are taken from the treatment plan for Case 3. It is assumed intrinsically in the method that follows that the patient’s previous radiotherapy treatment plans are available for review, including the DICOM files for the dose, structures, and CT dataset. In patients for whom previous treatment occurred elsewhere, all efforts should be made to securely obtain the required data, following appropriate information governance guidance for data transfer.

#### Datasets

The CT datasets for both the original and reirradiation plans are used in the methodology.

##### Original planning CT dataset

Once restored from the treatment planning archive, structures are generated that represent a range of isodoses in the patient’s original treatment plan. Isodose structures should range from the maximum dose in the plan down to the 50% isodose in 5 Gy increments and then 10 Gy increments down to the 10 Gy isodose. In order to account for the daily patient setup variations during the first treatment, the uncertainties in the delivered versus planned dose and the uncertainties in the registration process that follows, each isodose structure is expanded by a 5 mm planning organ at risk volume (PRV). Table 1 illustrates an example for a 65 Gy in 30 f previous treatment and includes suggested nomenclature for the isodose structures to group them alphabetically in the structure list.

**Table 1.** Isodose and PRV structures required for a previous 65 Gy radiation treatment.

Isodose level (Gy)	Structure name (isodose)	Structure name (PRV)
65	y65Gy	z65GyPRV
60	y60Gy	z60GyPRV
55	y55Gy	z55GyPRV
50	y50Gy	z50GyPRV
45	y45Gy	z45GyPRV
40	y40Gy	z40GyPRV
35	y35Gy	z35GyPRV
30	y30Gy	z30GyPRV
20	y20Gy	z20GyPRV
10	y10Gy	z10GyPRV

Abbreviation: PRV = planning organ at risk volume.

##### Co-Registration of datasets

The original dataset (CT_Orig) is co-registered to the new treatment planning dataset (CT_Irrad) using rigid image registration tools in the Eclipse TPS. When the patient’s position is variable between the two setups, registration match priority is given to the high dose region. Once co-registered, the PRV structures (Table 1) are copied from the CT_Orig dataset to the CT_Irrad.

##### Reirradiation planning CT dataset

With the previous treatment isodose PRV structures in place, delineation of the reirradiation clinical target volumes (CTVs) and OARs can proceed as normal. PTVs and PRVs for these are generated following standard treatment planning protocols.

#### Consideration of overlap and recovery

**Figure 1.**
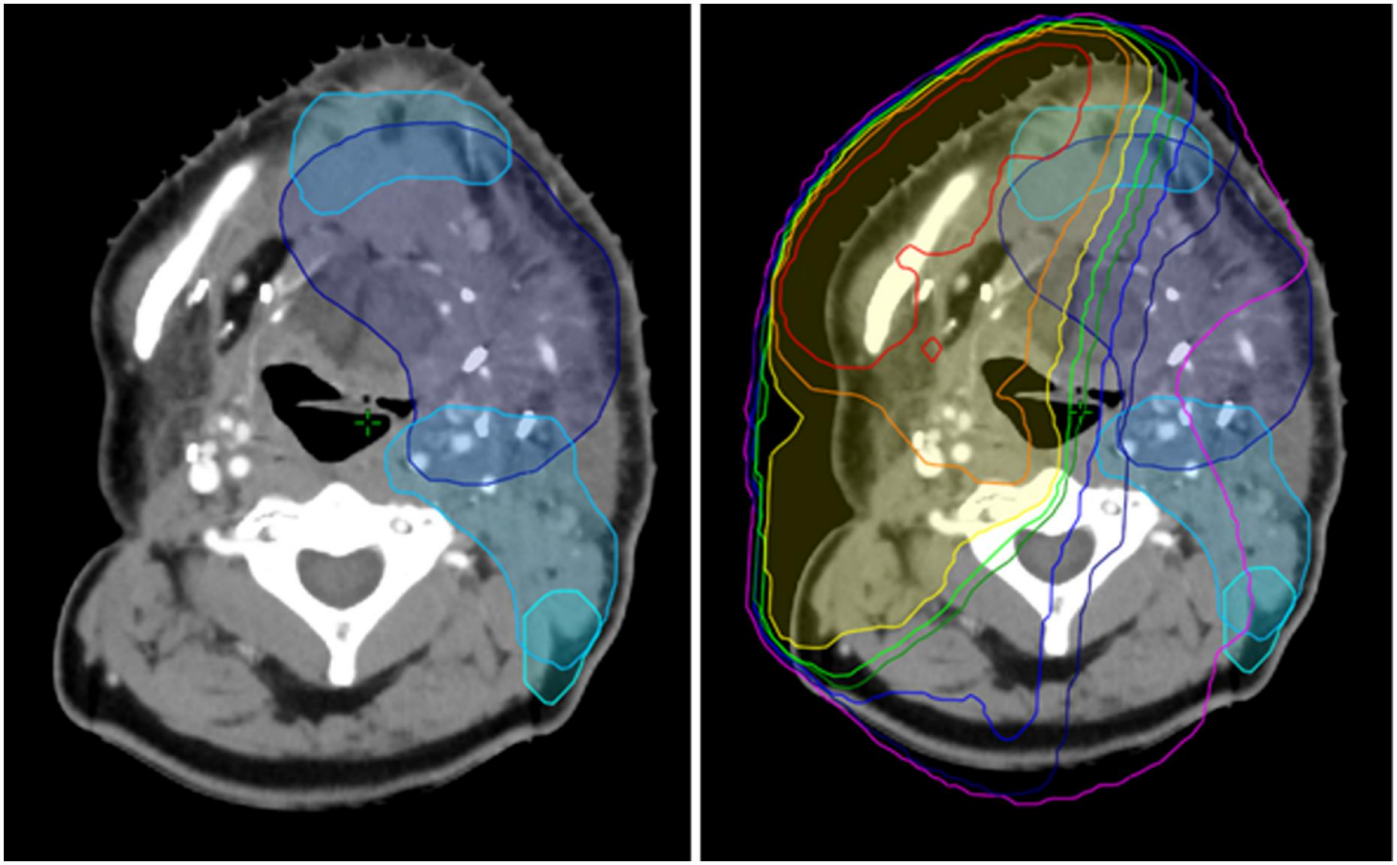
(Left) Intended PTVs for new treatment: 65 Gy (dark blue), 60 Gy (cobalt blue), 54 Gy (cyan). (Right) Overlay of PRV isodose contours from previous treatment (ranging from red to violet), highlighting areas of overlap with new treatment volumes. Abbreviations: PTVs = planning target volumes; PRV = planning organ at risk volume.

The CT_Irrad dataset is examined to check for overlap of the PRV isodose structures from the first treatment (Table 1) with the new intended PTVs (Figure 1). Consideration should be given to the potential for dosimetric recovery following the first radiotherapy treatment. The cumulative dose limit across the two courses of treatments should be jointly recommended by the clinicians and the multidisciplinary team. In all 3 cases discussed here, a 50% dosimetric recovery was assumed for the previous treatment isodoses.

By definition, an isodose contour of the *n*th isodose encloses all voxels receiving *at least n* Gy in dose. The maximum dose within the *n*th isodose contour is therefore dictated by the (*n* + 1)th isodose contour. An approximation of the mean dose within the *n*th isodose contour is simply the arithmetic mean of the *n*th and (*n* + 1)th isodose to which the 50% recovery assumption is then applied (Table 2).

**Table 2.** Worked example for determining the mean dose within each isodose contour including a 50% recovery assumption.

**Isodose structure** (*n*th isodose)	**Maximum dose in treatment 1** ((*n* + 1)th isodose)	**Mean dose in treatment 1** ([*n*+(*n* + 1)]/2)	Mean dose (50% recovery)
z65GyPRV	(Plan max dose, eg, 67 Gy)	66Gy	33Gy
z60GyPRV	65Gy	62Gy	31Gy
z55GyPRV	60Gy	58Gy	29Gy
z50GyPRV	55Gy	53Gy	26Gy
z45GyPRV	50Gy	48Gy	24Gy
z40GyPRV	45Gy	43Gy	21Gy
z35GyPRV	40Gy	38Gy	19Gy
z30GyPRV	35Gy	33Gy	16Gy
z20GyPRV	30Gy	25Gy	13Gy
z10GyPRV	20Gy	15Gy	8Gy

#### Creating optimization structures

Based on the advised maximum physical dose permitted across the two treatments, the maximum allowable dose (*D*)Gy within each overlapping isodose structure is determined for the new plan. Together with a minimum dose objective (*D-5)Gy*, these provide the necessary inputs to the TPS optimizer for the new plan (Table 3).

**Table 3.** Determination of the maximum dose allowed within each isodose structure with a maximum dose limitation from the combined treatments of 75 Gy.

Isodose structure	Mean dose received (50% recovery)	**Maximum dose to structure (*D)***(Using maximum physical dose allowed = 75Gy)	Minimum dose to structure for optimization (*D-5*)
z65GyPRV	33Gy	42Gy	37Gy
z60GyPRV	31Gy	44Gy	39Gy
z55GyPRV	29Gy	46Gy	41Gy
z50GyPRV	26Gy	49Gy	44Gy
z45GyPRV	24Gy	51Gy	46Gy
z40GyPRV	21Gy	54Gy	49Gy
z35GyPRV	19Gy	56Gy	51Gy
z30GyPRV	16Gy	59Gy	54Gy
z20GyPRV	13Gy	62Gy	57Gy
z10GyPRV	8Gy	67Gy	62Gy

**Figure 2.**
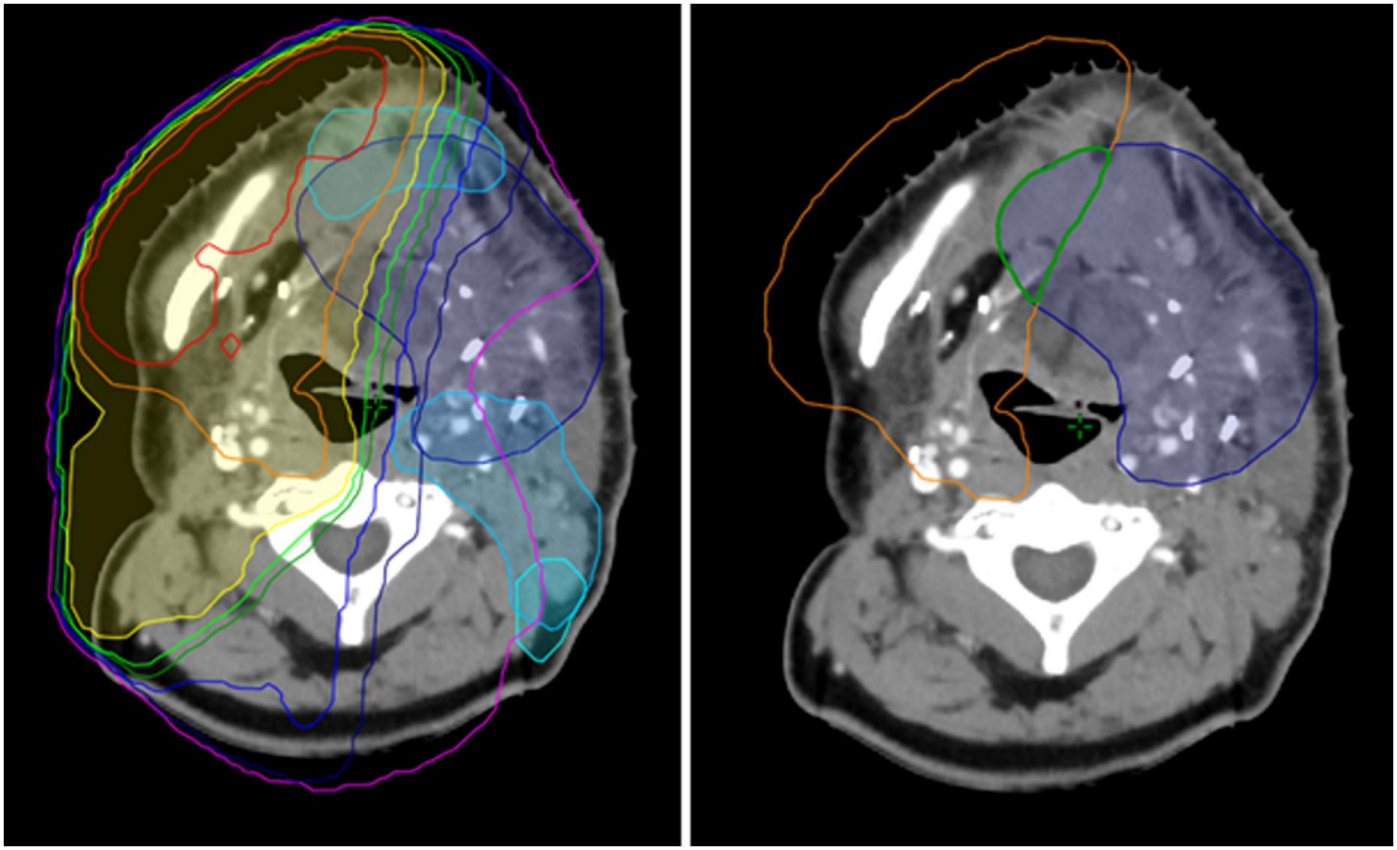
Example overlap region calculation for overlap of 55 Gy isodose with new PTV_6600.

**Figure 3.**
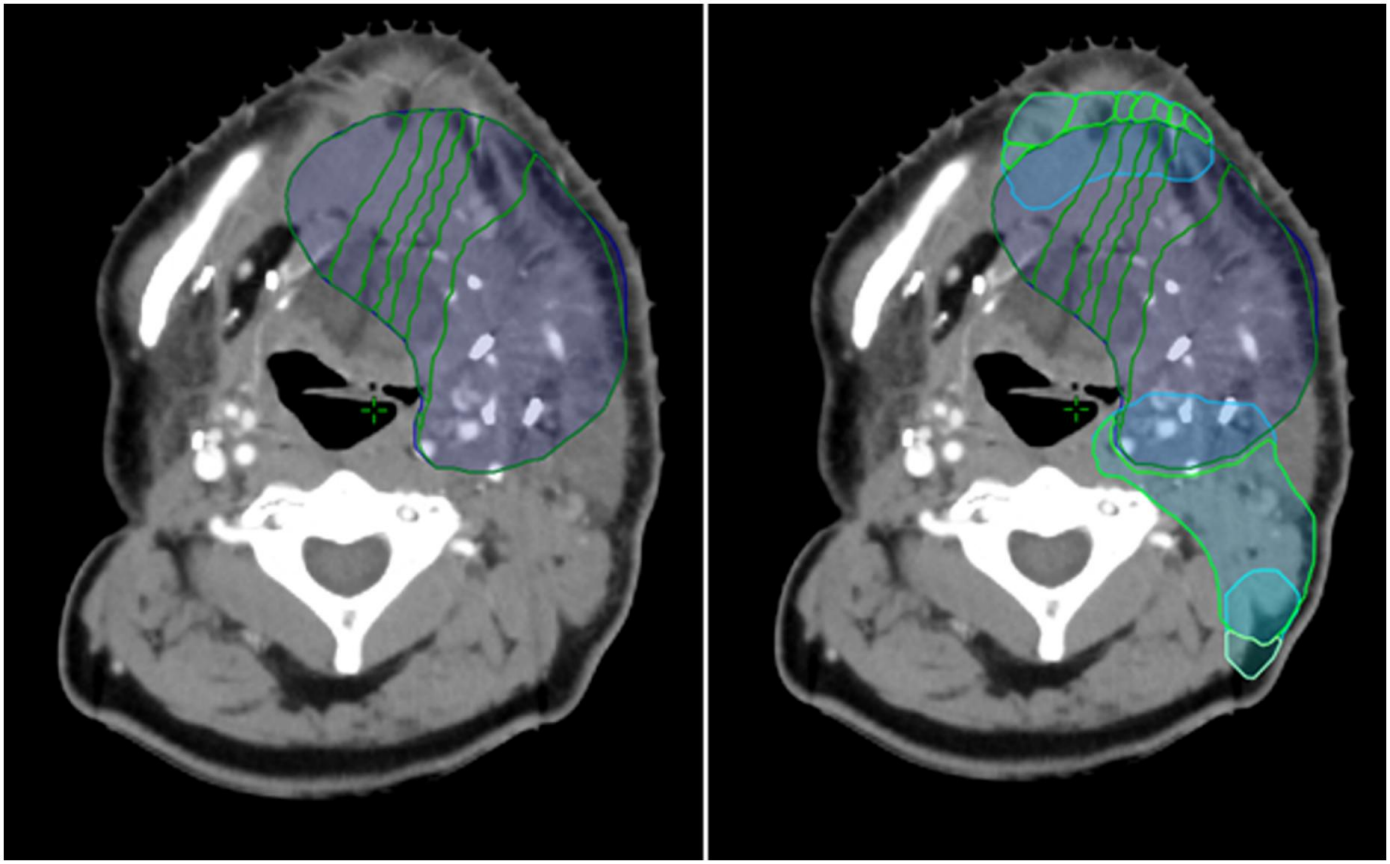
(Left) PTV_6600 split into a series of sub-PTVs (dark green), determined by overlap of previous isodose contours. (Right) Process repeated across all intended new target volumes. Abbreviation: PTVs = planning target volumes.

A series of Boolean operations now follows (which can be automated using the scripting features of a TPS) to split the target PTVs of the intended new treatment into a series of sub-PTVs based on their overlap with the isodose structures from the first treatment (Figure 2), and the maximum dose allowed (Table 3). Each sub-PTV is also cropped from its neighbour by a margin to avoid optimizer conflicts. This is repeated for all isodose overlaps (Figure 3, left) and for all intended new PTVs (Figure 3, right).

**Figure 4.**
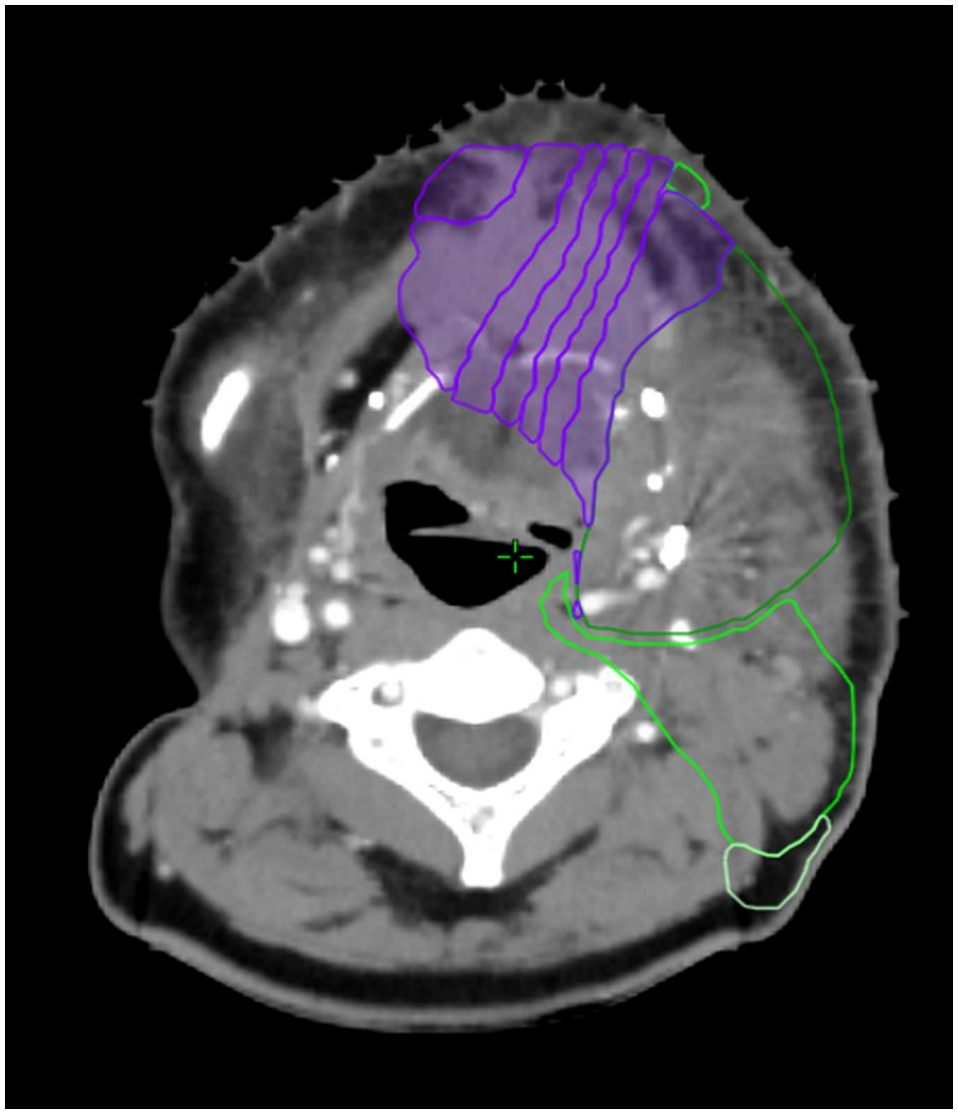
Sub-PTVs from multiple PTVs combined for simplicity (purple contours) and remaining areas of PTV unaffected by overlap (green contours). Abbreviation: PTVs = planning target volumes.

As each sub-PTV is created based only on its overlap with the previous isodose contours, sub-PTVs originating from separate PTVs can be combined to simplify the optimization to join sub-PTVs of equal dose targets. Areas of PTV not of concern for overlap can remain as separate optimization structures for their PTV (Figure 4).

#### OAR tolerances

Tolerance doses for the serial-like OARs such as spinal cord and brainstem are adjusted by accounting for the dose delivered to these organs from the first treatment using biologically effective dose (BED) formalism. Calculations for the new tolerance doses utilize the same 50% recovery assumptions and alpha/beta ratio values are taken from QUANTEC recommendations.^11^ Accounting for the doses from the first treatment and the 50% recovery assumption, the maximum allowed doses for the spinal cord (BED_total_ <120 Gy) and brainstem (BED_total_ <127 Gy) were calculated accordingly. The dose constraints for other OARs such as the carotid arteries (BED_total_ <120-140  Gy^1^^,^^3^), parotid glands and oral cavity, were set at the discretion of clinicians^2^ and were agreed in the established departmental peer review.^12^

#### Resulting dose distributions

**Figure 5.**
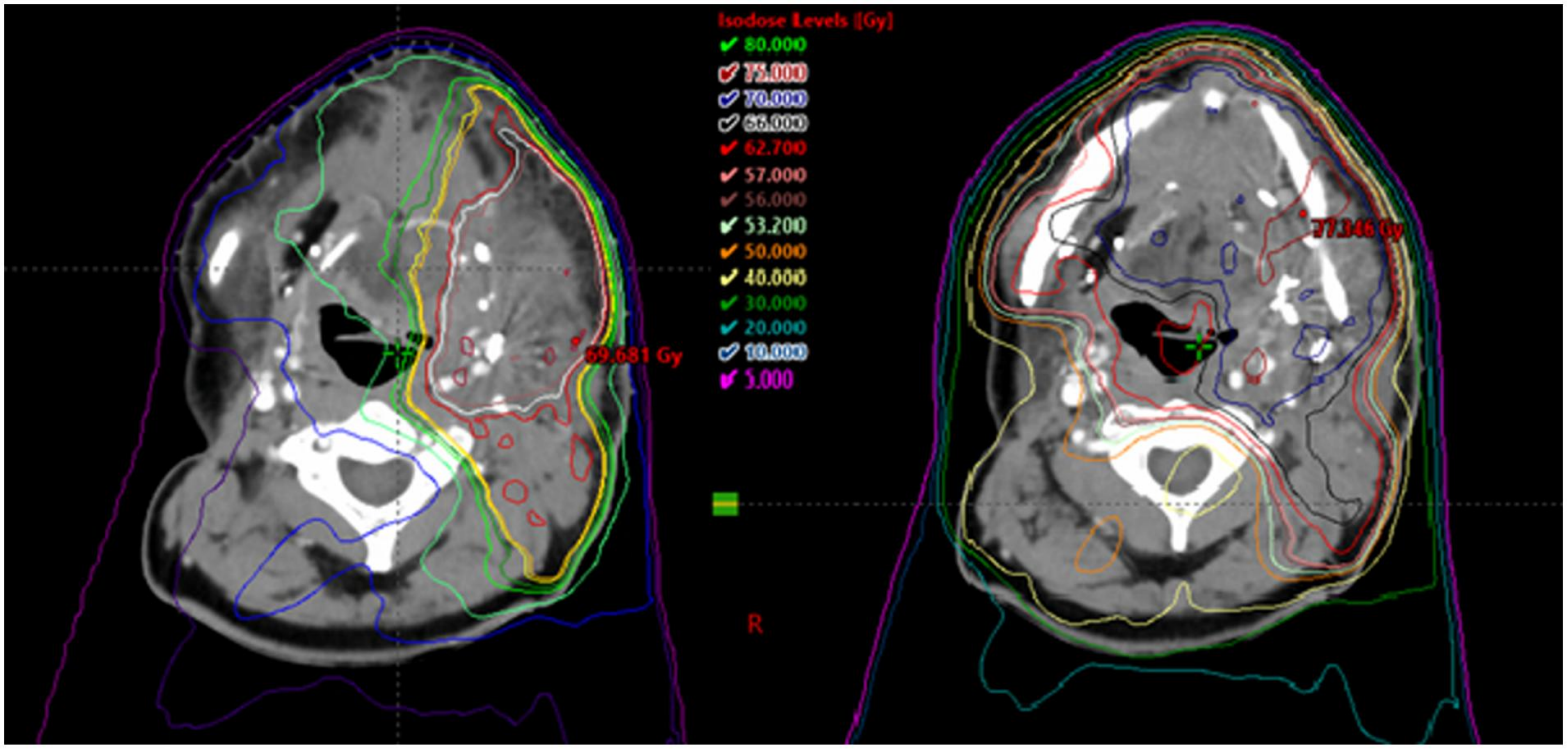
(Left) Planned dose distribution for new treatment and (right) composite dose distribution from both treatments, showing smooth junctioning of the two complex IMRT distributions. Abbreviation: IMRT: intensity modulated radiotherapy.

The resulting dose distribution should be reviewed both in consideration of the new radiotherapy treatment (Figure 5, left) and by producing a composite plan to show the combined dose from each treatment plan (Figure 5, right).

## Case studies outcomes

The reirradiation was tolerated with grade 2 radiation toxicities at worst for Case 1. At the time of writing, he had been disease free over 6½ years post-salvage surgery and reirradiation. As for Case 2, the reirradiation to the contralateral neck was tolerated well with grade 1 radiation toxicity only. She had also been disease free over 5½ years post-second neck dissection and reirradiation. Unfortunately for Case 3, the tumour recurred and progressed rapidly at the subcutaneous area and the skin during the chemo-reirradiation. The patient unfortunately died 3 months post-reirradiation.

## Discussion

The prognosis of oral cavity SCC with pENE is usually poor, with 1 year survival of 51% despite multi-modality treatment.^13^ This reirradiation technical note does not claim to provide any prognostic improvement but provides a methodology for a technique for which many potential patients of diverse clinical scenarios could benefit from. Head and neck cancer patients represent a highly heterogeneous group and there is no single best radiotherapy approach that fits all. Although the selected clinical cases all represent oral tongue cancer patients who had undergone prior primary surgery and adjuvant radiotherapy, all 3 patients were still heterogeneous. The ultimate treatment was recommended collectively via the MDT and after individualized discussions with the patients. The risk of contralateral neck metastasis with oral tongue cancer, in particular tumours of T3 classification or worse,^14^ is well recognized. Departmentally, it was not a routine procedure to offer upfront bilateral neck dissection for all patients with cT1-2 tumours. Despite good pre-operative clinical and radiological examinations, some primary tumours can be upstaged from clinically (c)T2 to pathological (p)T3 post-operatively. The adjuvant radiotherapy management of the neck can be complex with any pathological upstaging, with no single treatment consensus that could universally encompass all clinical scenarios.^15^ For that reason, certain patients might be offered personalized adjuvant radiotherapy. In the case of recurrence, despite well-intended treatment, this reirradiation methodology could provide the alternative support and flexibility.

The Case 1 patient had not initially received radiotherapy to the pathologically node negative neck (pN0) after the first surgery. There is no firm clinical consensus that a pN0 neck should be routinely irradiated post-operatively.^15^^,^^16^ Some institutions or oncologists may offer post-operative radiotherapy for pN0, due to concerns of the inability to deliver safe effective radical radiotherapy again if cancer recurs. Nevertheless, the morbidity of radiotherapy affecting patients’ quality of life should be considered, and there is currently a randomized study addressing irradiation for pN0 neck.^17^ Similarly, the same conundrum is applicable to the contralateral non-operated clinically cN0 neck, especially in patients with ipsilateral pN0 neck.^18^ Although it is acknowledged that patients like Cases 2 and 3 might have benefited from treatment (surgery or radiotherapy) to the contralateral cN0 neck,^7^ not all patients would be suitable or accept to undergo potentially morbid bilateral neck surgery, in addition to the postoperative radiation treatment. Importantly, as demonstrated by the 3 cases of different clinical scenarios, the methodology of this reirradiation technique was deliverable to all and may provide an alternative treatment option for other patients too.

One of the clinical uncertainties of reirradiation is the total dose required to achieve the radical/therapeutic aim. With this multiple dose avoidance technique, the total proportion of the CTVs receiving the prescribed dose is not necessarily 100%. Some areas of the CTVs will inevitably receive below the full prescribed dose. Caudell et al^4^ reported that doses of 50-66 Gy appear to be adequate in postoperative reirradiation after surgical removal of gross disease. In the cases presented here, the V50Gy of the high dose CTV varied significantly between the 3 patients (Table 4). Given the poorest outcome in our limited cohort was in Case 3, it is noteworthy that the V50Gy was lowest in this case and future work and studies should perhaps consider this parameter versus clinical outcomes. Clearly, more data are needed to determine the optimal post-operative radiation dose, but this reirradiation method could provide an alternative for patients who would not otherwise be routinely offered reirradiation after salvage surgery.

**Table 4.** Variation in V50Gy for the high dose CTV in 3 clinical cases of reirradiation.

Case	High dose CTV V50Gy
1	77.4% [CTVn_6500]
2	45.7% [CTVn_6500]
3	20.6% [CTVn_6600]

Abbreviation: CTV = clinical target volume.

A crucial clinical aspect of note is that all 3 cases were reirradiated with the assumption of 50% tissue recovery from previous radiation treatment. This was based on the assumption of 50% recovery in the central nervous system (CNS) organs used in some of the head and neck reirradiation studies.^2^^,^^5^ Indeed, this approach could be deemed radical on the OARs, as some reirradiation studies did not assume OAR dose recovery, necessitating compromises to the recurrent CTVs. There are numerous serial and parallel organs in the head and neck region with different biological properties. The lack of robust clinical and radiobiological data on radiation dose recovery of individual organ makes the clinical decision on the cumulative dose constraints of the recurrent cancer or OARs challenging. Indeed, the 50% recovery assumption of non-CNS soft tissues (eg, carotid vessels) for all 3 cases can be deemed over-cautious and perhaps contentious, especially when compromises of CTV coverage (consequently the therapeutic aim) were required. As evidenced in Case 3 (Table 4), the intention of avoiding late or catastrophic toxicities like carotid injury had led to considerable underdosing of the therapeutic CTV (V50Gy = 20%). There has been increasing evidence since these 3 cases were reirradiated, showing the relative safety of carotid arteries receiving BED_total_ <120.^1^^,^^3^^,^^19^ Strict observation of the 50% rule in tissue recovery could compromise the fundamental aim of reirradiation. Ultimately decision for reirradiation method is complex and there is always the pragmatic balance of cure and significant toxicities to be made. In all 3 cases, the dose-constraint decisions were real-world approaches and had been fully discussed in MDT and the patients. Fundamentally though, the demonstrated reirradiation methodology per se is independent of all the clinical uncertainties. This reirradiation planning pathway can be used once a multidisciplinary decision on the permissible dose constraints, however complex, is made.

The European Society for Radiotherapy and Oncology and European Organisation for Research and Treatment of Cancer recently released a consensus on reirradiation.^20^ This consensus was published a few years after our reirradiation technique was first applied. It was agreed that the overlap of irradiated volumes, rather than the overlap of target volumes (eg, PTV) or isodose lines, should be used when reirradiation is considered or delivered. This is because target volumes are based on treatment planning and the final delivered dose might not necessarily coincide with the planned dose distribution. The methodology presented here accounts for some of this uncertainty through the use of PRVs for the isodose structures from the first treatment. Nevertheless, the use of delivered rather than planned isodoses would potentially enhance the accuracy when accounting for dosimetric overlap. In the absence of a robust method for calculating accumulated delivered dose distributions, the technique presented here, based on planned isodose lines, can still be considered to guide the decision or delivery of reirradiation. This technique allows either a conservative or more drastic approach, depending on clinicians’ acceptance of the isodose uncertainties and the decision on the maximum permissible dose or cumulative dose limit.

The availability of dose accumulation software such as Varian Velocity now permits the final ‘delivered’ dose to be calculated using deformable image registrations of on-treatment cone beam CT imaging.^21^ This could aid the decision on the dose limit when it comes to reirradiation. However, such software is not readily available in all radiotherapy departments. Additional staffing resource would also be needed to commission and to ensure full accuracy of the deformable registration (eg, the mobile tongue, bolus on neck). This demonstrated reirradiation technique does not require any specific software but simply a planning system capable of performing rigid registration. A known limitation of this methodology, though, is the challenge with two treatment schedules with significantly different fraction sizes, for example, a previous accelerated or stereotactic radiation plan with now a cancer recurrence requiring hyperfractionated treatment. The direct dose accumulation from the registration of plans can become unreliable, and careful total BED calculation will therefore be required.

It is acknowledged that the 65 Gy in 30f regimen, although popular as a mildly hypofractionated definitive head and neck radiotherapy approach in the United Kingdom, is not a standard dose fractionation for reirradiation. In addition to the cumulative dose toxicities, late effects with reirradiation should be considered and many clinicians would limit the dose to no more than 2 Gy per fraction. If resource-allowed, hyperfractionation can and should be considered too.^22^ The dose fractionation management will require a separate discussion outside the scope of this technical note.

Increasing numbers of patients are likely to be considered for reirradiation. Though such a reirradiation method may be technically feasible, the long-term practicality of adopting such a treatment technique as standard practice requires consideration of the resource impacts to the clinical physics service. Plans of this nature require several days of planning and checking time which must be considered in the context of wider operational requirements. Automation of some treatment planning tasks through scripting can potentially reduce the resource impact (particularly through the creation of the sub-PTVs used for optimization) and the use of artificial intelligence tools can further improve resource provision. Consideration of QA requirements should also be given, as such plans are often complex and highly modulated which brings into question the delivery accuracy and capability of the treatment machines.

## Conclusion

A novel technique that uses isodose structures from previous treatments to create optimization volumes for dose avoidance in the new treatment can be used to create highly complex, tailored treatment plans to a range of clinical scenarios. Though technically feasible, careful patient selection and multidisciplinary decision-making on treatment objectives and constraints are crucial.
